# Cyto–Genotoxic Effect Causing Potential of Polystyrene Micro-Plastics in Terrestrial Plants

**DOI:** 10.3390/nano12122024

**Published:** 2022-06-12

**Authors:** Mandeep Kaur, Ming Xu, Lin Wang

**Affiliations:** 1 Henan Key Laboratory of Earth System Observation and Modelling, Henan University, Kaifeng 475004, China; mk9041985@gmail.com; 2 College of Geography and Environmental Science, Jinming Campus, Henan University, Kaifeng 475004, China; 3 BNU-HKUST Laboratory for Green Innovation, Beijing Normal University, Zhuhai 519088, China

**Keywords:** plastic, micro-plastic size, *Allium cepa* root chromosomal aberration assay, aberrations, chromosomal abnormality index, nuclear abnormality index

## Abstract

The polystyrene micro-plastics (Ps-MPs) is one of the leading pollutants found in both aquatic and terrestrial ecosystems. While most of the studies on the morphology and cyto-toxicity of MPs have been based on aquatic organisms, their effects on terrestrial plants are still scarcely known. The present study was an attempt to measure the effect of different sizes (80, 100, 200, 500, 1000, 2000, 4000, and 8000 nm) and concentrations (100 and 400 mg/L) of Ps-MPs on the root length and chromosomes of root tip cells of *Allium cepa* using *A. cepa* root chromosomal aberration assay. Large size Ps-MPs (4000 and 8000 nm) showed the highest reduction in *A. cepa* root length; however, the differences were not significant (at *p* ≤ 0.05), with respect to negative control (Milli-Q water). The mitotic index showed both significant size- and concentration-dependent decreases, being the lowest (12.06%) in 100 nm at 100 mg/L concentration, with respect to the control (25.05%). The chromosomal abnormality index (CAI) and nuclear abnormality index (NAI) showed significant decreases, with respect to negative control. In addition, the induction of micro-nucleated cells was also observed in *Allium* root tip cells, when treated with MPs of all sizes, which can predict direct DNA damage to the plant cells. Hence, we conclude that most of the MP sizes caused cyto-toxic and nuclear damage by adversely impacting the spindle formation and induction of micro-nucleated cells in *Allium cepa* root tip cells. To the best of our knowledge, this is the first study that showed the effect of considerable size range of Ps-MP sizes on the root length and cell division in plants.

## 1. Introduction

Plastic is omnipresent and has become one of the main components of our modern-day consumption, including food packaging, cosmetics, personal care, and textiles. Plastic has acquired importance due to its low manufacturing cost, light weight, bioavailability, flexibility, stability, durability, viscosity, and long-lasting ability [1,2]. The World Economic Forum [3] reported that, by 2050, there will be more plastic than fishes in the world’s oceans. Human consumption has generated more than 8 billion tons of plastic since 1950s, out of which, only 10% has been recycled, while the rest ended up directly in soil and ocean ecosystems [4].

Plastic is considered non-biodegradable, in general; however, due to various biological, chemical, and physical processes, larger size particles breakdown into smaller ones, which are known as micro-plastics (MPs). MPs with a size range of <5 μm cannot decompose easily or be collected for recycling; hence, they enter directly into the soil, water bodies, food chains, and air [5]. Being ubiquitous in nature and persistent in the environment for longer time, MPs have become a major concern as a pollutant [6,7]. Commercially, high- and low-density polypropylene (PP), polyvinylchloride (PVC), polyethylene (PE), polyethylene tere-phthalate (PET), and polystyrene (Ps) are the most extensively used plastics [8], out of which, polystyrene micro-plastic (Ps-MP) is a major threat as a pollutant in both terrestrial and aquatic ecosystems [9,10]. Most of the micro-plastics reach terrestrial environment either directly from daily-used plastic products and industrial abrasives or through the degradation and decomposition of the discarded/disposed plastics [11].

Over the last few decades, scientists and various governmental/non-governmental agencies extensively studied MP pollution, including its source of origin and harmful effects on the environment, especially the aquatic ecosystem. Many studies reported on the ingestion and accumulation of MP particles in the diverse organs of fishes, such as the liver, kidney, gut, and gills, as well as the detrimental effects on their overall health and survival [12,13,14]. Micro-sized plastic particles can more easily absorbed and mobilized, and their bio-accumulation may result in toxic effects [15]. Ps-MP accumulation in aquatic ecosystems has been shown to cause slower growth and disrupted reproduction in marine gastropod *Crepidula onyx* [16], smaller sized eggs and reduced hatching in species of copepod (*Calanus helgolandicus*) [17], and physical damage to zooplanktons [18,19]. A reduced number of larvae and smaller size adults in water flea (*Ceriodaphnia dubia*), due to MP fibers interference in swimming, was observed by Ziajahromi et al. [20], while a shorter lifespan in adult Pacific mole crabs (*Emerita analoga*) was reported by Horn et al. [21]. Researchers also studied the effects of MPs on animal cells and tissues, and they found that the accumulation of MPs caused inflammation in small intestines, lowered sperm count, and, as a result, fewer and smaller size off springs in mice [22,23]. Interestingly, Ragusa et al. [24] reported the presence of pigmented MP particles in human placenta for the first time, but the source in the bloodstream was not identified. Another study recently showed that in vivo polyethylene micro-plastics treatment significantly increased micro-nucleation, nucleo-plasmic bridge, and nuclear bud formation in human peripheral blood lymphocytes [25].

However, the fate and determination of MPs in soil is poorly known; however, the soil ecosystem acts as a long-term sink for plastic-based debris, and the majority of the plastics generated each year (−300 million tons) end up in the soil [26]. MP-derived waste was found to be 23 times higher in terrestrial ecosystems, as compared to aquatic ones [27]. Agricultural soils are more prone to being exposed to MPs via the fibers present in sewage sludge [28,29], plastic mulching [30], foams or fragments due to littering, street runoff [31], and wind deposition [32]. The MP particles travel from the soil surface to deeper layers, where they degrade to certain level and result in deposition. Yu et al. [33] reported that earthworms (*Lumbricus terrestris*) ingest and mobilize low density polyethylene (LDPE) MPs from the topsoil surface into the deeper layers, thus leaching the debris into ground water.

Previous reports suggested that MPs can be absorbed into the plant cells from the soil through the cellular barriers and these particles could be accompanied by other toxic pollutants, such as heavy metals, as well [34,35,36]. Indeed, MPs in the soil ecosystem are reported to interact with detrimental heavy metals, such as cadmium and mercury, and can serve as vectors for their uptake and transport into living organisms via the food chain [37,38,39]. In plants, heavy metals can generate high oxidative stress, resulting in cellular damage and the disruption of cellular ionic homeostasis, whereas their accumulation in animal body may cause alteration in the functioning of vital organs, such as the brain, heart, kidneys, liver, and bones [40].

The impact of MPs on living organisms, in general, is hard to interpret because MPs exist in varied shapes, sizes, and chemical compositions. Different sizes, shapes, and polymer morphology, as well as the mode of reactivity and high surface/volume ratio of MPs, can decrease or enhance its bioavailability within the open environment [41]. MPs with different particle sizes and shapes show varied effects on plant growth and development [42]. The absorption of MPs into a living cell depends on its size and shape, as the cell is surrounded by a size-selective barrier or cell wall on the outside and cell membrane on the inside [43]; however, MPs still can easily pass through such barriers, as they are at least 100 times smaller than a living plant cell, and the size varies from as small as a virus to as large as a pencil eraser [44]. Recently, a study showed the association and accumulation of 40 nm and 1 μm polystyrene negatively charged micro-plastic spheres at the root tip and cap cell surfaces in Arabidopsis (*Arabidopsis thaliana*) and wheat (*Triticum aestivum*); however, the study did not confirm MP presence inside the cell [44]. MPs have been shown to affect the overall health and development of plants. For example, Qi et al. [45] showed that the application of low-density polyethylene and starch-based biodegradable macro- and micro-sized plastic residues affected wheat growth, while the application of only micro-sized plastics showed more negative effects on both the vegetative and reproductive apparatus of wheat crop, as compared to the macro-plastics. In another study, the application of micro-sized polyethylene, polypropylene, and polystyrene plastic particles had a stronger negative impact on the vegetative growth of juvenile lime trees, as compared to macro-sized in a controlled pot experiment [46].

With plastics becoming a quintessential part of our daily lifestyle, there is an urgent necessity to assess the induced toxicity of MPs of all sizes and shapes on living cells [47]. For the management and risk assessment of polystyrene MPs, eco-toxicity tests employing plant-based models have been recommended by various national and international organizations, including the US Environmental Protection Agency (EPA),United Nations Environmental Program (UNEP), International Program on Plant Bioassay (IPPB), and World Health Organization (WHO) [48]. In order to understand the absorption and toxicity of variable-sized plastic particles on plants, in particular, in vivo models, such as the *A. cepa* root tip chromosomal aberration assay, would be best suited. The *A. cepa* assay is the preferred model for studying chromosomal aberrations because it provides better clarity of mitotic phases and the easy detection of chromosomal abnormalities, due to the low chromosome number (2 n = 16) and stability of karyotype. In addition, the inexpensiveness and easy availability of *A. cepa* bulbs throughout the year also make it an assay of choice [49]. The *A. cepa* root assay of cyto–genotoxicity of variable-sized Ps-MPs could provide a basic understanding that can be extrapolated in other plants [50,51,52].

Recently some studies have reported on the cyto–genotoxic effects of polystyrene MPs on plants such as *Vicia faba* and *Allium cepa* [43,52,53]. The effects of Ps-MPs of sizes 0.5 and 100 nm in *V. faba* reported an accumulation of 100 nm Ps-MPs into the root tissues of *V. faba*, which resulted in cyto–genotoxicity, such as micronucleus formation [43]. Another report by Maity et al. [52] measured the cyto–genotoxic nature of 100 nm Ps-MPs employing 25, 50, 100, 200, and 400 mg/L concentrations in *A. cepa* and confirmed the cyto–genotoxicity, in terms of different chromosomal (clumped chromosomes, laggard chromosomes, ring chromosomes, vagrant chromosomes, multi-polarity, sticky bridge, etc.) and nuclear aberrations. Additionally, Giorgetti et al. [53] reported on the cyto-toxicity of 50 nm size PS-MP nano-beads, which induced chromosomal abnormalities and micro-nucleated cells in *A. cepa* after MPs accumulation in the cytoplasmic and vacuolar parts of *A. cepa*.

Considering the toxicity of MPs in terrestrial plants, the present study evaluated the Ps-MPs induced effect on root length and cell division in *A. cepa*. Our study suggests that MPs, in general, have a negative impact on the root development and cell division of plants, and the severity of the impact can be influenced by the different sizes of MPs present in the plant’s vicinity. This work will lay down a foundation for using the *A. cepa* assay as risk assessment tool for detecting Ps-MP-induced cyto- and nuclear toxicity in higher plants. To the authors’ knowledge, this is the first report that included the effects of the considerable size ranges of Ps-MPs on the cell division of *A. cepa* root tip cells.

## 2. Materials and Methods

### 2.1. Model Plant

The *A. cepa* bulbs were purchased from the local vegetable market of Kaifeng city, Henan Province, China. Ps-MPs (80, 100, 200, 500, 1000, 2000, 4000, and 8000 nm; code number: 6-1-0005 to 6-1-0800) and analytical grade chemicals, such as orcein stain, working acids, and glassware, were purchased from Tianjin BaseLine ChromTech Research Centre, located in Tianjin city, China.

### 2.2. Ps-MPs Working Concentrations

Eight different sizes of Ps-MPs were selected for the present study. Two different working concentrations (100 and 400 mg/L) were chosen, based on the earlier studies [10,52]. Concentrations were adopted based on research from study of Maity et al. [52], where 100 and 400 mg/L concentrations of Ps-MPs showed significant cyto-toxic effects on the *A. cepa* root tip cells. The used concentrations may not represent the Ps-MPs contamination levels, in general, but possibly indicate the realistic concentrations in polluted areas. Ps-MPs working solutions were prepared from stock (procured as 250 mg, 10 mL) using Milli-Q water as a diluent. Prior to use, the solutions were properly homogenized by ultra-sonication for 30 min at 50 MHz.

Fresh, uniform, and equal-sized *Allium* bulbs with an average weight between 3.5 to 4 g and diameter 2 cm were selected, and dry roots were carefully removed with forceps, leaving behind the initial roots for fresh root growth. Experiment was carried out in triplicates (total of three denuded *Allium* bulbs); bulbs were incubated in MPs solutions, along with a negative control (Milli-Q water), in incubator for 72 h at 25 °C, with 50–60% humidity level. Time of incubation was selected based on many earlier studies, which proved the efficacy of *A. cepa* assay as one of the best short-term tests employed in environmental monitoring, providing satisfactory results within 72 h of treatment [49,54,55,56].

### 2.3. Experimentation

After incubation of *Allium* bulbs in Ps-MP solution, root length of each bulb was measured by using a hand ruler. The length of all roots in a single bulb was noted, and average root length was calculated. Cyto–genotoxic study was carried out using *A. cepa* root tip chromosomal aberration assay. For cyto–genotoxicity assessment, the collected roots were properly washed under tap water, followed by distilled water, and then fixed in Carnoy’s fluid for 12 h at 4 °C [56]. Subsequently, the fixed roots were transferred to 70% alcohol and stored in refrigerator at 4 °C temperature until use.

### 2.4. Microscopic Analysis

For the study of chromosomal abnormalities (CAs) and nuclear abnormalities (NAs), microscopic slides were prepared following the protocol of Sharma and Sharma [57]. Briefly, fixed root tips were dipped in 45% acetic acid for 5 min and acid hydrolyzed in watch glass containing 1 N HCl for 10 min using sprit lamp. Further, hydrolyzed roots were stained in 2% aceto-orcein solution for 30 min. The stained root tips were then placed in watch glass containing 2–3 drops of 45% acetic acid for few seconds to remove excess stain. After this, root tips were placed on clean glass slide, and the pointed root tip area was cut using a sharp blade; then, they were carefully covered with coverslip and squashed gently. Nine slides were scored for each MPs size, and a minimum of 1000 cells were scored per slide (10,000 cells/MP size) under a light microscope at 1000× magnification (Olympus CX31).

### 2.5. Calculations

The percent mitotic index (MI), phase index (PI), chromosomal abnormality index (CAI), and nuclear abnormality index (NAI) were calculated by using following formulae [52,58,59].

**Mitotic index**

$$\mathrm{MI}\;\left(\%\right)=\frac{\mathrm{Total}\;\mathrm{no}.\;\mathrm{of}\;\mathrm{dividing}\;\mathrm{cells}}{\mathrm{Total}\;\mathrm{no}.\;\mathrm{of}\;\mathrm{cells}\;\mathrm{observed}}\times 100$$

**Phase indices (PI)**

$$\;\mathrm{PI}\;\left(\%\right)=\frac{\mathrm{No}.\;\mathrm{of}\;\mathrm{cells}\;\mathrm{in}\;\mathrm{prophase}/\mathrm{metaphase}/\mathrm{anaphase}/\mathrm{telophase}}{\mathrm{Total}\;\mathrm{no}.\;\mathrm{of}\;\mathrm{cells}\;\mathrm{observed}}\times 100\;$$

**Chromosomal abnormality index**

$$\mathrm{CAI}\;\left(\%\right)=\frac{\mathrm{Total}\;\mathrm{no}.\;\mathrm{of}\;\mathrm{cells}\;\mathrm{with}\;\mathrm{chromosomal}\;\mathrm{abnormalities}}{\mathrm{Total}\;\mathrm{no}.\;\mathrm{of}\;\mathrm{dividing}\;\mathrm{cells}}\times 100\;$$

**Nuclear abnormality index**

$$\;\mathrm{NAI}\;\left(\%\right)=\frac{\mathrm{Total}\;\mathrm{no}.\;\mathrm{of}\;\mathrm{cells}\;\mathrm{with}\;\mathrm{nuclear}\;\mathrm{abnormalities}\;\left(\mathrm{MN}\;\&\;NB\right)}{\mathrm{Total}\;\mathrm{no}.\;\mathrm{of}\;\mathrm{dividing}\;\mathrm{cells}}\times 100\;$$

### 2.6. Statistical Analysis

Results have been presented as mean ± standard error (S.E.) of nine independent readings. Data were analyzed for the statistical significance between the mean difference of values and control group by one-way ANOVA and Tukey’s post-hoc test using SPSS software (ver. 20).

## 3. Results

**Figure 1:**
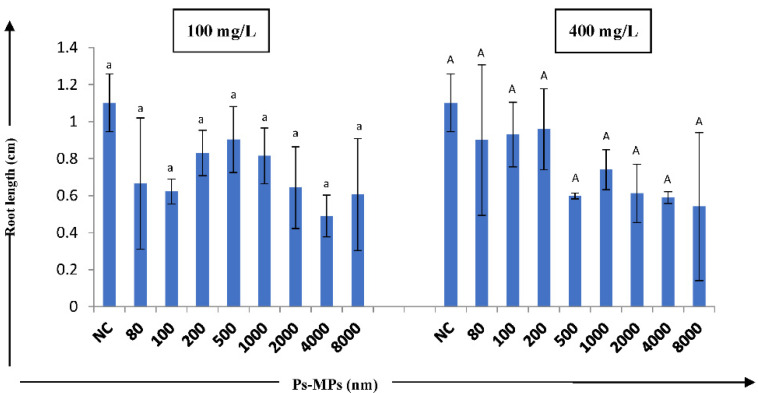
Effect of different sized Ps-MPs (at 100 and 400 mg/L concentrations) on root length (cm) of *A. cepa* bulbs. Results are shown as mean± S.E.; NC—negative control. The bars showing mean values of Ps-MPs contents at two concentrations having different letters (lowercase for 100 mg/L and uppercase for 400 mg/L) are significantly different at *p* ≤ 0.05 using one-way ANOVA; Tukey’s test.

The effect of Ps-MPs on *A. cepa* showed no significant decrease in root length at *p* ≤ 0.05, with respect to the negative control (Figure 1). 

No significant differences were observed in root length of *A. cepa* bulbs, but larger sized MPs showed the highest reduction in root length, as compared to the negative control, which could depict the toxic nature of large-sized MPs. At 100 mg/L concentration, as compared to the control (1.101 cm), a reduction in root length was observed in 4000 nm size Ps-MP (0.49 cm), while at 400 mg/L, the concentration reduction in the root length was found in 8000 nm-size Ps-MP (0.541 cm).

**Figure 2:**
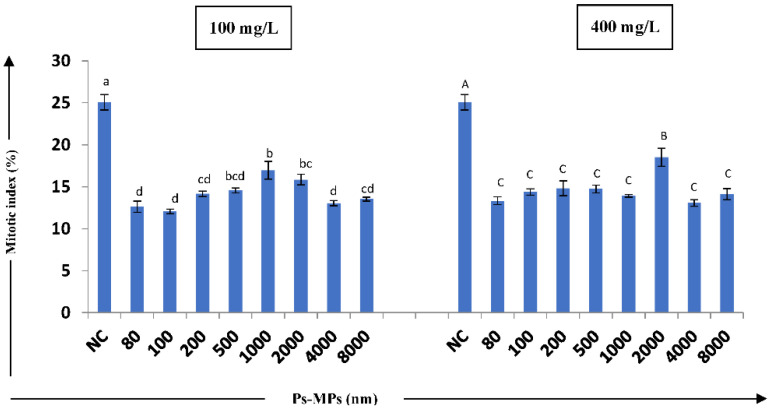
Effect of different sizes MPs (100 and 400 mg/L) on mitotic index (MI) (%) of *A. cepa* bulbs. Results are shown as mean± S.E.; NC—negative control. The bars showing mean values of Ps-MPs contents at two concentrations having different letters (lowercase for 100 mg/L and uppercase for 400 mg/L) are significantly different at *p* ≤ 0.05 using one-way ANOVA; Tukey’s test.

The cyto–genotoxic effect of Ps-MPs in the root tip cells of *A. cepa* was assessed by studying indices such as the mitotic, chromosomal, and nuclear abnormality indices. We observed a statistically significant decline in the mitotic index, in a size-dependent manner, for the tested Ps-MPs. The 100 nm-sized Ps-MPs showed a significant decrease in mitotic index (12.06 ± 0.284%) in the root tip cells at 100 mg/L concentration, as compared to the negative control (25.05 ± 0.917%) at *p* ≤ 0.05 after 72 h incubation (Figure 2 and Table 1). 

In the present study, phase indices were also calculated, as compared to the control, with decreases in the anaphase index (ARI) and telophase index (TLI); increases in the prophase index (PRI) and metaphase index (MTI) were observed in the root tip cells when treated with different sized Ps-MPs (Table 1).

To record the percentage of cyto-toxicity of the Ps-MPs in plants, the *A. cepa* root chromosomal aberration assay was used. The induction of diverse types of chromosomal aberrations (CAs) was reported in all the Ps-MP sizes, while no aberrations were detected in the negative control (Table 2).

**Figure 3:**
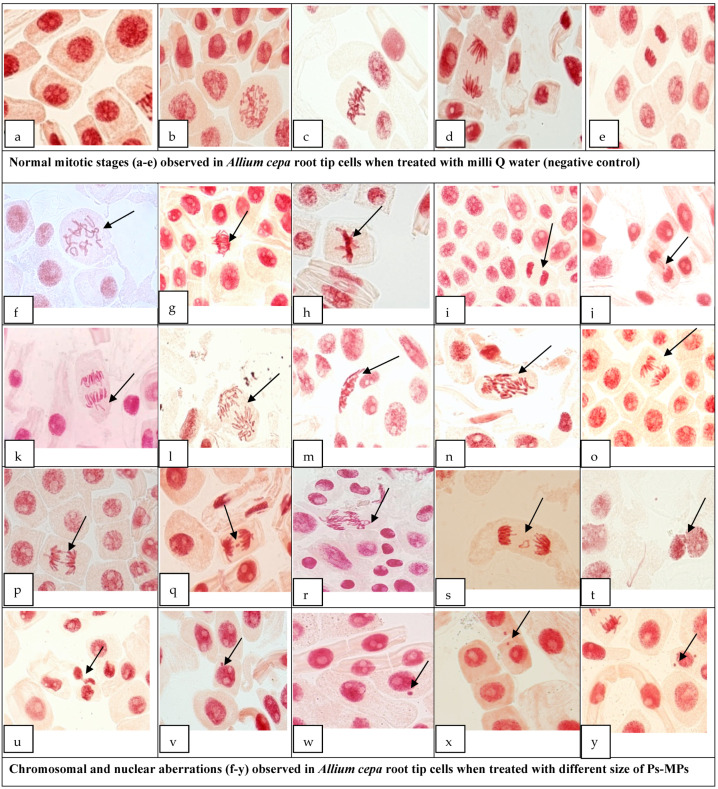
Representative pictures of normal mitotic phases, as well as chromosomal and nuclear aberrations in *A. cepa* root tip cells, following treatment with different sizes and concentrations of Ps-MPs, where: (**a**)—interphase; (**b**)—prophase; (**c**)—metaphase; (**d**)—anaphase; (**e**)—telophase; (**f**)—c-mitosis; (**g**)—delayed anaphases; (**h**)—metaphase clumped chromosome; (**i**)—anaphase clumped chromosome; (**j**)—laggard chromosomes; (**k**)—vagrant chromosome; (**l**)—distorted/disturbed anaphase; (**m**)—distorted/disturbed metaphase; (**n**)—multi-polarity; (**o**)—single bridge; (**p**)—double bridge; (**q**)—break; (**r**)—ring chromosome; (**s**)—ring chromosome; (**t**,**u**)—nuclear bud; (**v**,**w**)—single micronuclei cell; (**x**,**y**)—double micronuclei cell.

It was observed that the roots, when treated with a 100 mg/L concentration of 80 nm for 72 h, induced the highest percentage of c-mitosis (CM) (2.655%), while at 400 mg/L, delayed anaphase/s (DLA) (5.213%) and bridges (BG) (2.688%) were at maximum, as compared to the negative control. MPs of 100 nm size and a concentration of 400 mg/L showed the maximum percent of laggard chromosomes (LG) (1.002%) and distorted/disturbed anaphase/s (DSA) (1.907%), while 200 and 500 nm sizes of MPs at a 400 mg/L concentration showed clumped chromosomes (CC) (18.03%), vagrants (VG) (3.586%), multi-polarity (MP) (1.567%), and disorientation (DO) (1.815%) at the highest percentages, when compared to the control. Even largesized Ps-MPs viz. 1000 nm showed the induction of deleterious chromosomal abnormalities, such as ring chromosomes (RC)—(0.530%), while 2000 nm induced distorted/disturbed metaphase/s (DM)—3.431% and breaks (BK)—(1.013%) at 400 mg/L in the root tip cells. Overall, the present study showed the induction of different types of chromosomal aberrations, with the highest percent of CC followed by CM, DLA, VG, LG, distorted/disturbed metaphase (DM), DSA, MP, DO, BG, BK, and RC (Table 2). Some representative pictures of normal mitotic phases and chromosomal aberrations induced in *A. cepa* root tip cells, following the treatment of different sized Ps-MPs were shown in Figure 3. 

**Figure 4:**
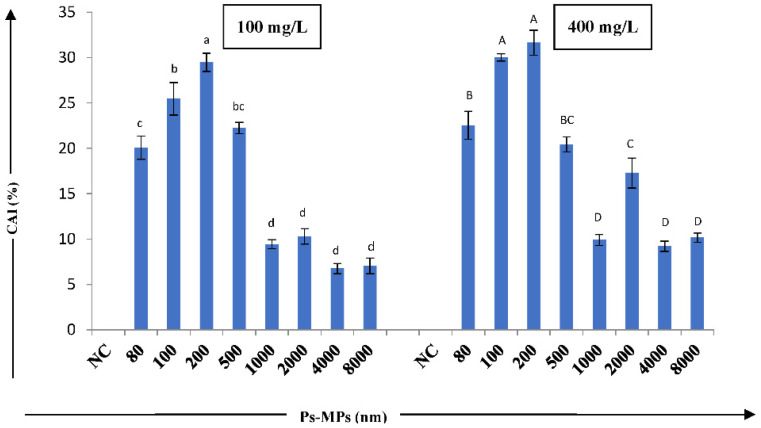
Effect of different sizes MPs (100 and 400 mg/L) on chromosomal abnormality index (CAI) (%) of *A. cepa* bulbs. Results are shown as mean± S.E.; NC—negative control. The bars showing mean values of Ps-MPs contents at two concentrations having different letters (lowercase for 100 mg/L and uppercase for 400 mg/L) are significantly different at *p* ≤ 0.05 using one-way ANOVA; Tukey’s test.

The Ps-MPs induced cyto–genotoxicity was observed by calculating the chromosomal abnormality index (CAI). In our study, CAI showed a statistically significant (at *p* ≤ 0.05) size-dependent decrease, where 200 nm-sized Ps-MP showed the highest CAI (31.65%), and the least was observed in 4000 nm MP (6.759%)-treated *Allium* root tip cells after 72 h of incubation (Figure 4). 

**Figure 5:**
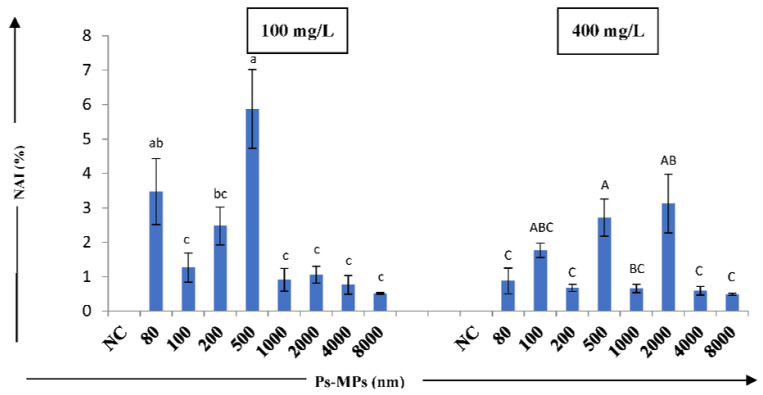
Effect of different sizes MPs (100 and 400 mg/L) on nuclear abnormality index (NAI) (%) of *A. cepa* bulbs. Results are shown as mean ± S.E.; NC—negative control. The bars showing mean values of Ps-MPs contents at two concentrations having different letters (lowercase for 100 mg/L and uppercase for 400 mg/L) are significantly different at *p* ≤ 0.05 using one-way ANOVA; Tukey’s test.

We also detected two types of nuclear abnormalities (NAs), namely micronuclei (MN) and nuclear bud (NB), in our test samples, whereas no NAs were detected in the negative control. In the present study, variations in the occurrence of MN and NB in the tested sizes and concentrations of MPs were found to be statistically significant, as compared to the negative control (Figure 5 and Table 2).

The maximum percentage of MN (2.43%) was observed in root tip cells when treated with Ps-MP of size 2000 nm at 400 mg/L, while NB (4.93%) recorded the highest percentage for 500 nm at 100 mg/L concentration. NBs were observed in all sizes of MPs, except for 8000 nm at 100 mg/L and 4000 nm at 400 mg/L after 72 h incubation, indicating the prominent cellular toxicity of Ps-MPs on the root tip cells of *A. cepa*. Overall, 100 mg/L concentration of the studied Ps-MPs showed high cyto-toxic effect, while 400 mg/L concentration induced nuclear abnormalities in the *Allium* root tip cells.

## 4. Discussion

The ubiquitous nature of MPs in the environment endangers the terrestrial ecosystem to a great extent. MPs occur in soil in different forms; their bioavailability to plants and soil organisms increases with size and depends on soil characteristics such as particle size and density, abundance/co-occurrence, chemical characteristics, etc. [60,61]. The present study planned to measure the effects of different sizes and concentrations of Ps-MPs on root length and plant root tip cell chromosomes. The insignificant reduction in *A. cepa* root growth was observed when treated with large-sized MPs; this might be due to the direct contact of larger-sized MPs with the root pores, which blocked and hindered the root apical meristem activity. It is known that the larger-sized Ps-MP particles cannot easily enter into the plant cell and, therefore, are adsorbed to the root surfaces, thereby inhibiting the uptake and absorption of water, as well as the essential nutrients required for growth and development in plants [62]. Bosker et al. [63] found that MPs of 4.8 µm size can accumulate in the pores of seed capsule of *Lepidium sativum* (cress) and significantly reduce the germination rate and root growth after 8 and 24 h exposure, respectively. Researchers have reported on the accumulation of different size MPs on the root surfaces of alfalfa and rice [64], *Vicia faba* [43], *A. cepa* [52], and wheat [44]. The particle size of MP acts as an important factor in determining their interaction with living tissues and cells; hence, their accumulation and distribution [65].

Increase in the cyto–genotoxicity of any toxicant can be recognized by the decline in the mitotic index of the cells [66]. In the present study, the significant size- and concentration-dependent decrease in the mitotic index was observed, with respect to the control. Gopinath et al. [1], in their study, attributed the decrease in the MI of *A. cepa* root cells to the potential of MPs in DNA synthesis inhibition, abduction of mitotic phases, and slow cell progression. A similar cytotoxic effect of 100 nm-sized Ps-MPs at 100 mg/L concentration was reported earlier by Maity et al. [53] and Jiang et al. [43]. Many earlier studies have reported on the significant toxicity of different sized MPs on the normal mitotic cell cycle of plants [67,68].

MPs were found to result in mito-depressive effects in plants by blocking the G2 phase of the normal mitotic cell cycle. This can further lead to the inhibition of regulators and DNA replication impairment, which can cause disturbances in the prophase index [52,69,70]. In the present study, phase indices were calculated to investigate the inhibition of mitotic cell division and increases in the prophase index (PRI) and metaphase index (MTI), while decreases in the anaphase (ARI) and telophase index (TLI) of *A. cepa* were observed when treated with different sizes and concentrations of Ps-MPs. The G2 phase inhibition of the cell cycles of the root tip cells can disturb the normal prophase index [52], but this was not significant in our study.

CAI results indicate the high cyto–genotoxic potential of small- and large-sized Ps-MPs. The cyto–genotoxicity can be attributed to a hindrance in DNA and protein synthesis. Gopinath et al. [1] observed that MPs can interrupt the nucleic acid metabolism, thus affecting protein and DNA synthesis, resulting in a range of chromosomal and nuclear abnormalities. Hindrances in nucleic acid and protein synthesis can alter the cell/nucleus volume, producing giant nucleus cells [71]. Many reports have indicated significant genotoxic effects of MPs on wheat [43], ryegrass [72], *V. faba* [44], spring onion [73], and *A. cepa* [52]. Maity et al. [52] reported significant fluctuations in MI and the induction of various chromosomal aberrations in *A. cepa* root tip cells when treated with different concentrations (25, 50, 100, 200, and 400 mg/L) of 100 nm-sized Ps-MPs. The underlying mechanism behind the genotoxic effects of MPs is largely unknown, but it might be due to the accumulation of MPs in the root tissues, thereby blocking cell wall pores and disrupting the transport of essential nutrients [44].

Irrespective of the size and concentration used in the present study, we observed the highest percentages of CC, CM, DLA, VG, BG, BK, etc. Chromosomal aberrations lead to destabilization of the genome, resulting in various genotoxic and mutagenic effects, including disturbances or malformation in spindle fibers, chromosome movement failure, breakage and fusion of chromosomes/chromatids, chromosomal pole shifting due to microtubule depolymerization, and alteration in the activation of the enzymes necessary for replication [49,52,74,75].The induction of c-mitosis can be due to hindrance in disulfide bonds formation, which effects the tubulin organization required for spindle microtubules [51,52]. Subsequently, laggard chromosomes can arise due to spindle disturbances or failure in anaphasic and pro-metaphasic movement [76,77], whereas clumped or sticky chromosomes can be formed due to the faulty functioning of specific non-histonic proteins, which are important for chromatid segregation, as well as chromosomal organization and separation [78,79]. Vagrants may arise due to the action of external agent’s c-mitotic force, resulting in the movement of chromosome away from the anaphasic pole or equatorial plate [80]. Chromosomal breaks are formed due to the unfinished or mis-repaired DNA molecules required for linear continuity of the chromosome’s structure [81], while the anaphasic bridge results from chromosomal breakage, stickiness, fusion, para-centric inversion, and altered functioning of the replication enzymes [82]. The ring chromosome can arise due to breaks in the chromosome arms and fusion of the proximal broken ends, which leads t oa loss of the distal material or rings; they can be formed by telomere dysfunction, triggering the fusion of reactive chromosome ends [52,83,84].

Ps-MPs also induced the formation of NB and MN in the *A. cepa* root tip cells, which mainly results from acentric or lagging chromatid fragments or a whole chromosome that is not present in the daughter nuclei after mitosis. These lagging fragments are not able to correctly attach to the spindle during chromosomes segregation in anaphase stage of mitosis; thus, they are unable to pass to the daughter nuclei [85]. These microstructures are enclosed by a nuclear membrane, and they are structurally similar but smaller in size, as compared to the main nuclei [86]. MN formation indicates the genotoxicity of MPs, due to DNA damage, hence the chromosomal instability. The formation of NB in *A. cepa* root tip cells might be due to the direct breaking action of the Ps-MPs on the genetic material or inhibition of spindle fibers formation [50,87]. The action of MPs on root tip cells may lead to cytokinesis inhibition, resulting in the formation of MN cells [88]. From the present study, it comes into sight that the impacts of different sizes of micro-plastics are varied and can be dependent on the type of plant, type and size of the plastics, concentration, and different experimental conditions.

## 5. Conclusions

The present study indicated cyto–genotoxic potential of Ps-MP particles in *A. cepa* root tip cells by induction of high percentage of chromosomal and nuclear aberrations. The study also suggests the suitability of *A. cepa* root chromosomal aberration as a reliable cyto–genotoxicity test, as *A. cepa* showed common basic genetic constitution throughout the eukaryotic organisms; the results can be helpful in the extrapolation of data in other higher animals and mammals used as test models. Our study is the first report based on the toxic effects of considerable size ranges of Ps-MPs on *A. cepa* root tip cells. Both large- and small-sized MPs showed the morpho-toxicity, cyto–genotoxicity, and induction of nuclear abnormalities in *A. cepa* root tip cells by reduction in the root growth, mitotic index, and induction of high percentage of chromosomal and nuclear abnormalities. The occurrence of clastogenic aberrations in the present study suggests the chromosomal breakage and spindle disrupting potential of Ps-MPs in terrestrial plants, irrespective of their size and concentration. Based on the results of the present study, environmental risk assessment and proper management of plastic debris of any type, size, and concentration are required before disposal, in order to avoid detrimental effects on the target, as well as non-target, living organisms. In the future, more research needs to be carried out, in order to address the knowledge gaps regarding the co-toxicity potential of MPs and other pollutants, as well as the mode of toxicity caused by MPs on plants, which will be of great importance.

## Figures and Tables

**Table 1:** Effect of different sizes and concentrations of Ps-MP on percentage of different mitotic phase indices and mitotic index.

Ps-MPs Size (nm)	Conc. (mg/L)	TNC	IC	PRI (% ± S.E.)	MTI (% ± S.E.)	ANI (% ± S.E.)	T LI (% ± S.E.)	MI (% ± S.E.) *
NC	0	12264	9199	39.38 ± 0.666	27.45 ± 0.755	23.49 ± 1.148	9.683 ± 0.935	25.05 ± 0.917
**80**	100	13036	11409	35.89 ± 1.649	24.26 ± 1.070	10.96 ± 1.848	4.730 ± 0.766	12.62 ± 0.656
	400	13697	11881	33.04 ± 0.963	28.92 ± 0.967	10.98 ± 0.882	4.467 ± 0.395	13.32 ± 0.458
**100**	100	14402	12671	35.89 ± 1.222	23.32 ± 0.592	10.41 ± 0.859	5.376 ± 0.344	12.06 ± 0.284
	400	12655	10842	34.27 ± 0.792	19.35 ± 0.843	8.424 ± 0.733	6.169 ± 0.463	14.38 ± 0.372
**200**	100	12584	10815	33.75 ± 1.388	19.01 ± 1.130	7.549 ± 0.444	7.306 ± 0.600	14.15 ± 0.332
	400	13042	11241	31.93 ± 1.181	19.40 ± 0.797	10.53 ± 0.441	6.107 ± 0.381	14.82 ± 0.875
**500**	100	12257	10478	34.95 ± 0.812	20.81 ± 0.885	9.166 ± 0.660	6.951 ± 0.461	14.56 ± 0.304
	400	12737	10877	38.26 ± 1.353	20.84 ± 1.375	8.8 ± 0.86500	9.235 ± 0.411	14.73 ± 0.446
**1000**	100	12825	10677	39.98 ± 1.556	26.94 ± 1.056	17.29 ± 1.170	6.053 ± 0.605	16.96 ± 1.068
	400	13518	11638	45.01 ± 1.397	25.24 ± 0.709	13.87 ± 0.923	5.550 ± 0.797	13.91 ± 0.162
**2000**	100	11781	9924	35.89 ± 1.418	27.41 ± 1.402	19.88 ± 0.698	6.16 ± 0.5460	15.84 ± 0.631
	400	6857	5625	46.26 ± 2.004	15.85 ± 1.634	13.69 ± 1.949	5.555 ± 0.864	18.51 ± 1.067
**4000**	100	14417	12548	41.40 ± 2.082	29.36 ± 2.244	16.28 ± 1.291	5.869 ± 0.650	13.04 ± 0.316
	400	15046	12904	42.79 ± 0.989	28.36 ± 0.502	13.62 ± 1.048	5.758 ± 0.503	13.06 ± 0.385
**8000**	100	13426	11611	41.94 ± 1.268	30.99 ± 1.281	15.01 ± 1.512	6.242 ± 0.920	13.53 ± 0.243
	400	13751	11812	45.33 ± 1.146	26.58 ± 1.142	12.45 ± 0.845	5.149 ± 0.791	14.13 ± 0.669

Ps-MPs—polystyrene micro-plastic; TNC—total number of cells; IC—interphase cells; NC—negative control; PRI—prophase index; MTI—metaphase index; ANI—anaphase index; TLI—telophase index; MI—mitotic index. * Mean values in columns are significantly different using one-way ANOVA at *p* ≤ 0.05.

**Table 2:** Effect of different sizes and concentrations of Ps-MP on percentage (%) of various chromosomal and nuclear abnormalities in *A. cepa* root tip cells.

MP-Ps Size (nm)	Conc. (mg/L)	CM (%)	DLA (%)	CC (%)	VG (%)	LG (%)	DM (%)	DSA (%)	MP (%)
NC	0	ND	ND	ND	ND	ND	ND	ND	ND
**80**	100	2.655 ± 0.465	1.299 ± 0.378	7.184 ± 0.678	1.739 ± 0.278	0.712 ± 0.083	3.063 ± 0.442	1.291 ± 0.405	0.736 ± 0.103
	400	0.591 ± 0.112	5.213 ± 0.674	8.032 ± 0.908	1.936 ± 0.218	0.467 ± 0.048	2.115 ± 0.334	1.497 ± 0.208	0.505 ± 0.013
**100**	100	1.173 ± 0.156	2.984 ± 0.405	11.86 ± 0.926	1.731 ± 0.212	0.967 ± 0.223	2.155 ± 0.364	1.182 ± 0.202	0.699 ± 0.172
	400	1.689 ± 0.200	2.02 ± 0.2120	15.41 ± 0.583	1.978 ± 0.312	1.002 ± 0.082	2.22 ± 0.1750	1.907 ± 0.260	1.268 ± 0.149
**200**	100	1.344 ± 0.166	2.999 ± 0.518	14.24 ± 0.859	2.276 ± 0.217	0.968 ± 0.195	1.649 ± 0.184	1.572 ± 0.161	1.009 ± 0.114
	400	1.054 ± 0.142	3.674 ± 0.531	18.03 ± 0.770	3.586 ± 0.291	0.931 ± 0.049	1.473 ± 0.223	1.304 ± 0.286	0.82 ± 0.2040
**500**	100	1.203 ± 0.317	1.807 ± 0.331	9.607 ± 0.608	1.565 ± 0.280	0.947 ± 0.188	1.576 ± 0.292	1.639 ± 0.143	1.116 ± 0.148
	400	ND	2.118 ± 0.284	10.33 ± 1.146	1.257 ± 0.192	0.796 ± 0.187	0.880 ± 0.242	1.218 ± 0.339	1.567 ± 0.514
**1000**	100	1.016 ± 0.269	2.066 ± 0.422	2.591 ± 0.472	0.978 ± 0.206	0.408 ± 0.000	1.165 ± 0.208	0.766 ± 0.197	0.501 ± 0.006
	400	1.363 ± 0.247	2.223 ± 0.360	2.562 ± 0.378	1.15 ± 0.2400	ND	0.922 ± 0.189	1.025 ± 0.143	0.504 ± 0.007
**2000**	100	1.385 ± 0.440	2.577 ± 0.359	3.405 ± 0.543	1.123 ± 0.155	0.877 ± 0.000	1.371 ± 0.262	1.016 ± 0.113	0.447 ± 0.009
	400	2.161 ± 0.345	3.421 ± 0.495	4.241 ± 0.549	2.277 ± 0.362	0.819 ± 0.125	3.431 ± 0.637	1.003 ± 0.159	1.515 ± 0.000
**4000**	100	0.456 ± 0.000	1.639 ± 0.352	2.063 ± 0.407	0.971 ± 0.083	0.944 ± 0.263	1.123 ± 0.267	0.918 ± 0.153	0.513 ± 0.000
	400	0.841 ± 0.144	2.069 ± 0.389	2.518 ± 0.276	1.288 ± 0.138	0.68 ± 0.1360	1.101 ± 0.213	0.67 ± 0.1400	0.462 ± 0.019
**8000**	100	1.012 ± 0.568	1.763 ± 0.270	2.099 ± 0.510	0.751 ± 0.157	0.764 ± 0.288	1.169 ± 0.324	1.378 ± 0.390	0.467 ± 0.000
	400	1.127 ± 0.173	2.32 ± 0.181	3.02 ± 0.471	0.868 ± 0.199	0.587 ± 0.132	0.64 ± 0.1050	0.828 ± 0.161	0.462 ± 0.030
**MP-Ps** **size (nm)**	**Conc.** **(mg/L)**	**DO (%)**	**BG (%)**	**BK (%)**	**RC (%)**	CAI (%) *	**MN (%)**	**NB (%)**	NAI (%) *
**NC**	**0**	**ND**	**ND**	**ND**	**ND**	**ND**	**ND**	**ND**	**ND**
**80**	100	0.926 ± 0.140	1.291 ± 0.248	0.698 ± 0.075	ND	20.08 ± 1.294	1.732 ± 0.432	2.238 ± 0.766	3.472 ± 0.952
	400	0.752 ± 0.170	2.688 ± 0.235	0.725 ± 0.307	ND	22.54 ± 1.529	0.513 ± 0.026	1.087 ± 0.000	0.875 ± 0.378
**100**	100	1.591 ± 0.288	1.499 ± 0.195	0.510 ± 0.000	0.508 ± 0.00	25.46 ± 1.782	1.106 ± 0.368	0.853 ± 0.222	0.843 ± 0.424
	400	1.054 ± 0.113	2.118 ± 0.184	0.485 ± 0.000	ND	30.02 ± 0.411	0.882 ± 0.158	1.385 ± 0.428	1.763 ± 0.521
**200**	100	1.757 ± 0.231	1.697 ± 0.299	0.540 ± 0.000	ND	29.47 ± 1.009	1.175 ± 0.211	1.635 ± 0.374	1.924 ± 0.549
	400	1.523 ± 0.192	1.642 ± 0.275	ND	ND	31.65 ± 1.380	0.640 ± 0.171	0.487 ± 0.029	0.376 ± 0.106
**500**	100	1.346 ± 0.201	1.693 ± 0.240	0.861 ± 0.141	ND	22.24 ± 0.633	1.698 ± 0.964	4.93 ± 0.9770	5.873 ± 1.149
	400	1.815 ± 0.313	1.306 ± 0.153	0.490 ± 0.005	0.461 ± 0.00	20.44 ± 0.830	1.388 ± 0.557	1.846 ± 0.472	2.412 ± 0.540
**1000**	100	0.683 ± 0.150	0.821 ± 0.150	ND	ND	9.429 ± 0.496	0.507 ± 0.000	0.741 ± 0.184	0.303 ± 0.330
	400	1.246 ± 0.210	1.191 ± 0.196	ND	0.53 ± 0.029	9.892 ± 0.601	0.627 ± 0.173	0.69 ± 0.2140	0.438 ± 0.124
**2000**	100	0.797 ± 0.129	0.651 ± 0.103	0.555 ± 0.099	0.416 ± 0.00	10.3 ± 0.8480	0.88 ± 0.1040	0.563 ± 0.083	0.704 ± 0.247
	400	0.869 ± 0.000	1.003 ± 0.253	1.013 ± 0.143	ND	17.29 ± 1.645	2.43 ± 0.5890	1.127 ± 0.407	2.081 ± 0.855
**4000**	100	0.487 ± 0.018	2.072 ± 1.479	ND	0.502 ± 0.00	6.759 ± 0.562	0.683 ± 0.186	0.497 ± 0.029	0.338 ± 0.273
	400	0.565 ± 0.095	0.635 ± 0.085	ND	ND	9.210 ± 0.563	0.588 ± 0.127	ND	0.261 ± 0.128
**8000**	100	0.528 ± 0.013	1.111 ± 0.219	ND	ND	7.05 ± 0.8750	0.511 ± 0.019	ND	0.171 ± 0.019
	400	0.461 ± 0.020	1.18 ± 0.214	0.469 ± 0.000	0.417 ± 0.00	10.16 ± 0.503	0.467 ± 0.028	0.555 ± 0.000	0.217 ± 0.030

NC—negative control; ND—not detected; CM—c-mitosis; DLA—delayed anaphases; CC—clumped chromosomes; VG—vagrant chromosome; LG—laggard chromosomes; DM—distorted/disturbed metaphase; DSA—distorted/disturbed anaphase; MP—multi-polarity; DO—disorientation; BG—bridge; BK—break; RC—ring chromosome; CAI—chromosomal abnormality index; MN—micronuclei cell; NB—nuclear bud; NAI—nuclear abnormality index.* Mean values in columns are significantly different using one-way ANOVA at *p* ≤ 0.05.

## Data Availability

All related data are provided in the manuscript.

## References

[1] Gopinath P.M., Saranya V., Vijayakumar S., Meera M.M., Ruprekha S., Kunal R., Pranay A., Thomas J., Mukherjee A., Chandrasekaran N. (2019). Assessment on interactive prospectives of nano-plastics with plasma proteins and the toxicological impacts of virgin, coronated and environmentally released-nanoplastics. Sci. Rep..

[2] Chamas A., Moon H., Zheng J., Qiu Y., Tabassum T., Jang J.H., Abu-Omar M., Scott S.L., Suh S. (2020). Degradation rates of plastics in the environment. ACS Sustain. Chem. Eng..

[3] World Economic Forum (2016). The New Plastics Economy: Rethinking the Future of Plastics.

[4] (2019). Consumer Reports. You Are Literally Eating Micro-Plastics. How You Can Cut Down Exposure to Them. The Washington Post..

[5] Rhodes C.J. (2018). Plastic pollution and potential solutions. Sci. Prog..

[6] Verla A.W., Enyoh C.E., Verla E.N. (2019). Microplastics, an emerging concern: A review of analytical techniques for detecting and quantifying microplatic. Anal. Methods Environ. Chem. J..

[7] Verla A.W., Enyoh C.E., Verla E.N., Nwarnorh K.O. (2019). Micro-plastic toxic chemical interaction: A review study on quantified levels, mechanism and implications. SNAppl. Sci..

[8] Bouwmeester H., Hollman P.C., Peters R.J. (2015). Potential health impact of environmentally released micro- and nanoplastics in the human food production chain. Environ. Sci. Technol..

[9] Plastic Europe (2018). Plastics–the Facts 2018. An Analysis of European Plastics Production, Demand and Waste Data.

[10] Zhu B.K., Fang Y.M., Zhu D., Christie P., Ke X., Zhu Y.G. (2018). Exposure of nanoplastics disturbs the gut microbiome in the soil oligochaete *Enchytraeus crypticus*. Environ. Pollut..

[11] Loos C., Syrovets T., Musyanovych A., Mailänder V., Landfester K., Nienhaus G.U., Simmet T. (2014). Functionalized polystyrene nanoparticles as a platform for studying bio-nano interactions. Beilstein. J. Nano Technol..

[12] Rodriguez-Seijo A., Lourenço J., Rocha-Santos T., Da Costa J., Duarte A., Vala H., Pereira R. (2017). Histo-pathological and molecular effects of micro-plastics in *Eisenia Andrei* Bouché. Environ Pollut..

[13] Zhang G.S., Liu Y.F. (2018). The distribution of microplastics in soil aggregate fractions in southwestern China. Sci. Total Environ..

[14] Huang F.Y., Yang K., Zhang Z.X., Su J.Q., Zhu Y.G., Zhang X. (2019). Effects of microplastics on antibiotic resistance genes in estuarine sediments. Acta Sci. Circum..

[15] Forte M., Iachetta G., Tussellino M., Carotenuto R., Prisco M., Falco M.D., Laforgia V., Valiante S. (2016). Polystyrene nanoparticles internalization in human gastric adenocarcinoma cells. Toxicol. Vitr..

[16] Lo H.K.A., Chan K.Y.K. (2018). Negative effects of microplastic exposure on growth and development of *Crepidula onyx*. Environ Pollut..

[17] Cole M., Lindeque P., Fileman E., Halsband C., Galloway T.S. (2015). The impact of polystyrene microparticles on feeding, function and fecundity in the marine copepod *Calanus helgolandicus*. Environ. Sci. Technol..

[18] Botterell Z.L.R., Beaumont N., Dorrington T., Steinke M., Thompson R.C., Lindeque P.K. (2019). Bioavailability and effects of microplastics on marine zooplankton: A review. Environ. Pollut..

[19] Yong M.M.H., Leistenschneider C., Miranda J.A., Paler M.K., Legaspi C., Germanov E., Araujo G., Burkhardt-Holm P., Erni-Cassola G. (2021). Microplastics in fecal samples of whale sharks (*Rhincodon typus*) and from surface water in the Philippines. Micro. Nano..

[20] Ziajahromi S., Kumar A., Neale P.A., Leusch F.D.L. (2017). Impact of microplastic beads and fibers on water flea (*Ceriodaphnia dubia*) survival, growth, and reproduction: Implications of single and mixture exposures. Environ. Sci. Technol..

[21] Horn D.A., Granek E.F., Steele C.L. (2019). Effects of environmentally relevant concentrations of microplastic fibers on Pacific mole crab (*Emerita analoga*) mortality and reproduction. Limnol. Ocean. Lett..

[22] Park E.-J., Han S., Park E.-J., Seong E., Lee G.-H., Kim D.-W., Son H.-Y., Han H.-Y., Lee B.-S. (2020). Repeated-oral dose toxicity of polyethylene microplastics and the possible implications on reproduction and development of the next generation. Toxicol. Lett..

[23] Jin H., Ma T., Sha X., Liu Z., Zhou Y., Meng X., Chen Y., Han X., Ding J. (2021). Polystyrene microplastics induced male reproductive toxicity in mice. J. Hazard Mater..

[24] Ragusa A., Svelato A., Santacroce C., Catalano P., Notarstefano V., Carnevali O., Papa F., Rongioletti M.C.A., Baiocco F., Draghi S. (2021). Plasticenta: First evidence of microplastics in human placenta. Environ. Int..

[25] Cobanoglu H., Belivermis M., Sikdokur E., Kilic O., Cayir A. (2021). Genotoxic and cytotoxic effects of polyethylene microplastics on human peripheral blood lymphocytes. Chemosphere.

[26] Pathan S.I., Arfaioli P., Bardelli T., Ceccherini M.T., Nannipieri P., Pietramellara G. (2020). Soil pollution from micro- and nanoplastic debris: A hidden and unknown biohazard. Sustainability.

[27] Horton A.A., Svendsen C., Williams R.J., Spurgeon D.J., Lahive E. (2017). Large microplastic particles in sediments of tributaries of the River Thames, UK-Abundance, sources and methods for effective quantification. Mar. Pollut. Bull..

[28] Wang Y., Wu C., Xue Q., Hui X. (2019). Effects of plastic contamination on water evaporation and desiccation cracking in soil. Sci. Total Environ..

[29] Li Q., Feng Z., Zhang T., Ma C., Shi H. (2020). Microplastics in the commercial seaweed nori. J. Hazard Mater..

[30] Steinmetz Z., Wollmann C., Schaefer M., Buchmann C., David J., Tröger J., Muñoz K., Frör O., Schaumann G.E. (2016). Plastic mulching in agriculture. Trading short-term agronomic benefits for long-term soil degradation. Sci. Total Environ..

[31] Bläsing M., Amelung W. (2018). Plastics in soil: Analytical methods and possible sources. Sci. Total Environ..

[32] Brahney J., Mahowald N., Prank M., Cornwell G., Klimont Z., Matsul H., Prather K.A. (2021). Constraining the atmospheric limb of the plastic cycle. Proc. Nat. Acad. Sci. USA.

[33] Yu M., Van Der Ploeg M., Lwanga E.H., Yang X., Zhang S., Ma X., Ritsema C.J., Geissen V. (2019). Leaching of microplastics by preferential flow in earthworm (*Lumbricus terrestris*) burrows. Environ. Chem..

[34] Bandmann V., Müller J.D., Köhler T., Homann U. (2012). Uptake of fluorescent nanobeads into BY2-cells involves clathrin dependent and clathrin-independent endocytosis. FEBS Lett..

[35] Zhu F., Changyin Z., Chao W., Gu C. (2019). Occurrence and ecological impacts of microplastics in soil systems: A Review. Bull.Environ. Contam. Toxicol..

[36] Campanale C., Massarelli C., Savino I., Locaputo V., Uricchio V.F. (2020). A detailed review study on potential effects of microplastics and additives of concern on human health. Int. J. Environ. Res. Public Health.

[37] Barboza L.G.A., Vieira L.R., Branco V., Figueiredo N., Carvalho F., Carvalho C., Guilhermino L. (2018). Microplastics cause neurotoxicity, oxidative damage and energy-related changes and interact with the bioaccumulation of mercury in the European seabass, *Dicentrarchuslabrax* (Linnaeus, 1758). Aquat. Toxicol..

[38] Banaee M., Soltanian S., Sureda A., Gholamhosseini A., Haghi B.N., Akhlaghi M., Derikvandy A. (2019). Evaluation of single and combined effects of cadmium and micro-plastic particles on biochemical and immunological parameters of common carp (*Cyprinus carpio*). Chemosphere.

[39] Bradney L., Wijesekara H., Palansooriya K.N., Obadamudalige N., Bolan N.S., Ok Y.S., Rinklebe J., Kim K.H., Kirkham M.B. (2019). Particulate plastics as a vector for toxic trace-element uptake by aquatic and terrestrial organisms and human health risk. Environ. Int..

[40] Singh R., Gautam N., Mishra A., Gupta R. (2011). Heavy metals and living systems: An overview. Indian J. Pharmacol..

[41] Boyle K., Ormeci B. (2020). Microplastics and nanoplastics in the freshwater and terrestrial environment: A review. Water.

[42] Rillig M.C., Lehmann A., Machado de Souza A.A., Yang G. (2019). Microplastic effects on plants. New Phyto..

[43] Jiang X., Chen H., Liao Y., Ye Z., Li M., Klobucar G. (2019). Ecotoxicity and genotoxicity of polystyrene microplastics on higher plant *Vicia faba*. Environ. Pollut..

[44] Taylor S., Pearce C., Sanguinet K., Hu D., Chrisler W., Kim Y., Wang Z., Flury M. (2020). Polystyrene nano- and microplastic accumulation at Arabidopsis and wheat root cap cells, but no evidence for uptake into roots. Environ. Sci. Nano..

[45] Qi Y., Yang X., Pelaez A.M., Lwanga H.E., Beriot N., Gertsen H., Garbeva P., Geissen V. (2018). Macro- and micro- plastics in soil-plant system: Effects of plastic mulch film residues on wheat (*Triticum aestivum*) growth. Sci. Total Environ..

[46] Verla A.W., Enyoh C.E., Obinna I.B., Verla E.N., Qing W., Chowdhury Md A.H., Enyoh E.C., Chowdhury T. (2020). Effect of macro-and micro-plastics in soil on growth of Juvenile Lime Tree (*Citrus aurantium*). AIMS Environ. Sci..

[47] Smith M., Love D.C., Rochman C.M., Neff R.A. (2018). Microplastics in seafood and the implications for human health. Curr. Environ. Health Rep..

[48] Silva G.H., Monteiro R.T.R. (2017). Toxicity assessment of silica nanoparticles on *Allium cepa*. Ecotoxicol. Environ. Contam..

[49] Kaur M., Bhatti S.S., Soodan R.K., Katnoria J.K., Bhardwaj R., Nagpal A.K., Xu M. (2021). Physico-chemical characterization of agricultural soil samples and their modulatory effects on cytogenetic and biochemical parameters of *Allium cepa*. J. Soil Sci. Plant Nutr..

[50] Datta S., Singh J., Singh J., Singh S., Singh S. (2018). Assessment of genotoxic effects of pesticide and vermicompost treated soil with *Allium cepa* test. Sustain. Environ. Res..

[51] Verma S., Srivastava A. (2018). Morphotoxicity and cytogenotoxicity of pendimethalin in the test plant *Allium cepa* L.—A biomarker based study. Chemosphere.

[52] Maity S., Chatterjee A., Guchhait R., Deb S., Pramanick K. (2020). Cytogenotoxic potential of a hazardous material, polystyrene microparticles on *Allium cepa* L.. J. Hazard Mater..

[53] Giorgetti L., Span_o C., Muccifora S., Bottega S., Barbieri F., Bellani L., Ruffini Castiglione M. (2020). Exploring the interaction between polystyrene nanoplastics and *Allium cepa* during germination: Internalization in root cells, induction of toxicity and oxidative stress. Plant Physiol. Biochem..

[54] Levan A. (1938). The effect of colchicine on root mitosis in *Allium*. Hereditas.

[55] Leme D.M., Marin-Morales M.A. (2009). *Allium cepa* test in environmental monitoring: A review on its application. Mutat. Res. Rev. Mutat. Res..

[56] Sabeen M., Mahmood Q., Bhatti Z.A., Faridullah, Irshad M., Bilal M., Hayat T., Irshad U., Akbar T.A., Arslan M. (2020). *Allium cepa* assay based comparative study of selected vegetables and the chromosomal aberrations due to heavy metal accumulation. Saudi J. Biol. Sci..

[57] Sharma A.K., Sharma A. (1980). Chromosome Techniques: Theory and Practice.

[58] Hemachandra C.K., Pathiratne A. (2015). Assessing toxicity of copper, cadmium and chromium levels relevant to discharge limits of industrial effluents into inland surface waters using common onion, *Allium cepa* bioassay. Bull. Environ. Contam. Toxicol..

[59] Hemachandra C.K., Pathiratne A. (2017). Cytogenotoxicity screening of source water, wastewater and treated water of drinking water treatment plants using two in vivo test systems: *Allium cepa* root based and Nile tilapia erythrocyte based tests. Water Res..

[60] Atuanya E.I., Udochukwu U., Dave-Omoregie A.O. (2016). Bioavailability and Toxicity of Plastic Contaminants to Soil and Soil Bacteria. Br. Microbiol. Res. J..

[61] Scherer C., Brennholt N., Reifferscheid G., Wagner M. (2017). Feeding type and development drive the ingestion of microplastics by freshwater invertebrates. Sci. Rep..

[62] D’Aquino L., Depinto M.C., Nardi L., Morgana M., Tommasi F. (2009). Effect of some light rare earth elements on seed germination, seedling growth and antioxidant metabolism in *Triticum durum*. Chemosphere.

[63] Bosker T., Bouwman L., Brun N., Behrens P., Vijver M. (2019). Microplastics accumulate on pores in seed capsule and delay germination and root growth of the terrestrial vascular plant *Lepidium sativum*. Chemosphere.

[64] Miralles P., Johnson E., Church T.L., Harris A.T. (2012). Multiwalled carbon nanotubes in alfalfa and wheat: Toxicology and uptake. J. R. Soc. Interface.

[65] Lin S., Reppert J., Hu Q., Hudson J.S., Reid M.L., Ratnikova T.A., Rao A.M., Luo H., Ke P.C. (2009). Uptake, translocation, and transmission of carbon nano-materials in rice plants. Small.

[66] Pérez-de-Luque A. (2017). Interaction of nanomaterials with plants: What do we need for real applications in agriculture?. Front. Environ. Sci..

[67] Smaka-Kincl V., Stegnar P., Lovka M., Toman M.J. (1996). The evaluation of waste, surface and ground water quality using the *Allium* test procedure. Mutat. Res..

[68] Fernandes T.C.C., Mazzeo D.E.C., Marin-Morales M.A. (2007). Mechanism of micronuclei formation in polyploidizated cells of *Allium cepa* exposed to trifluralin herbicide. Pestic. Biochem. Phys..

[69] Singh D., Roy B.K. (2017). Evaluation of malathion-induced cytogenetical effects and oxidative stress in plants using *Allium* test. Acta Physiol. Plant.

[70] Kumari M., Khan S.S., Pakrashi S., Mukherjee A., Chandrasekaran N. (2011). Cytogenetic and genotoxic effects of zinc oxide nanoparticles on root cells of *Allium cepa*. J. Hazard Mater..

[71] Sharma S., Vig A.P. (2012). Genotoxicity of atrazine, avenoxan, diuron and quizalofop-pethyl herbicides using the *Allium cepa* root chromosomal aberration assay. Terr. Aquat. Environ. Toxicol..

[72] Oh N., Park J.H. (2014). Endocytosis and exocytosis of nanoparticles in mammalian cells. Int. J. Nanomed..

[73] Boots B., Russell C.W., Green D.S. (2019). Effects of micro-plastics in soil ecosystems: Above and below ground. Sci. Technol. Environ..

[74] Machado A.A.S., Kloas W., Zarfl C., Hempel S., Rillig M.C. (2018). Microplastics as an emerging threat to terrestrial ecosystems. Glob. Chang. Biol..

[75] Fatma F., Verma S., Kamal A. (2018). Monitoring of morphotoxic, cytotoxic and genotoxic potential of mancozeb using *Allium* assay. Chemosphere.

[76] Rajeshwari A., Roy B., Chandrasekaran N., Mukherjee A. (2016). Cytogenetic evaluation of gold nanorods using *Allium cepa* test. Plant Physiol. Biochem..

[77] Gaulden M.E. (1987). Hypothesis: Some mutagens directly alter specific chromosomal proteins (DNA topoisomerase II and peripheral proteins) to produce chromosome stickiness, which causes chromosome aberrations. Mutagenesis.

[78] Potapova T., Gorbsky G.J. (2017). The consequences of chromosome segregation errors in mitosis and meiosis. Biology.

[79] Fiskesjö G. (1979). Mercury and selenium in a modified *Allium* test. Hereditas.

[80] Evans H.J., Scott D., Bridges B.A., Sobels F.H. (1977). Molecular mechanism in the induction of chromosome aberrations. Progress in Genetic Toxicology.

[81] Panda K.K., Achary V.M.M., Krishnaveni R., Padhi B.K., Sarangi S.N., Sahu S.N., Panda B.B. (2011). *In vitro* biosynthesis and genotoxicity bioassay of silver nanoparticles using plants. Toxicol. Vitro..

[82] Gisselsson D. (2001). Ring Chromosomes: Vicious Circles at the End and Beginning of Life.

[83] Raghuvanshi S.S., Singh A.K. (1976). Effect of gamma rays on growth and karyokinetic activity in *Trigonella foenum-graecum L*. Cytologia.

[84] Vicars H., Karg T., Warecki B., Bast I., Sullivan W. (2021). Kinetochore-independent mechanisms of sister chromosome separation. PLoS ONE Genet..

[85] Kwon M., Leibowitz M.L., Lee J.H. (2020). Small but mighty: The causes and consequences of micronucleus rupture. Exp. Mol. Med..

[86] Fenech M., Chang W.P., Volders M.K., Holland N., Bonassi S., Zeiger E. (2003). Human project: Detailed description of the scoring criteria for the cytokinesis block micronucleus assay using isolated human lymphocyte cultures. Mutat. Res..

[87] Fernandes T.C.C., Mazzeo D.E.C., Marin-Morales M.A. (2009). Origin of nuclear and chromosomal alterations derived from the action of an aneugenic agent e trifluralin herbicide. Ecotoxicol. Environ. Saf..

[88] Nefic H., Musanovic J., Metovic A., Kurteshi K. (2013). Chromosomal and nuclear alterations in root tip cells of *Allium cepa* L. Induced by alprazolam. Med. Arch..

